# Seven-Year Durability of Improvements in Urinary Incontinence After Roux-en-Y Gastric Bypass and Sleeve Gastrectomy

**DOI:** 10.1001/jamanetworkopen.2022.46057

**Published:** 2022-12-09

**Authors:** Wendy C. King, Amanda S. Hinerman, Leslee L. Subak

**Affiliations:** 1 Department of Epidemiology, Graduate School of Public Health, University of Pittsburgh, Pittsburgh, Pennsylvania; 2 Department of Obstetrics and Gynecology, Stanford University School of Medicine, Stanford, California; 3 Department of Urology, Stanford University School of Medicine, Stanford, California

## Abstract

This cohort study evaluates the durability of improvements in urinary incontinence among women and men who underwent Roux-en-Y gastric bypass (RYGB) or sleeve gastrectomy.

## Introduction

Obesity is an independent risk factor for urinary incontinence (UI).^1^ Bariatric surgery is an effective treatment for severe obesity^2^ and reduces the frequency and prevalence of UI among both women and men.^1,3^ However, using follow-up data through 3 years, the Longitudinal Assessment of Bariatric Surgery 2 (LABS-2) study reported that initial improvements in UI diminish after 1 to 2 years.^4^ Longer-term follow-up is scarce.^1,3^ Using LABS-2 follow-up data through 7 years, this cohort study evaluates the durability of improvements in UI among women and men who underwent Roux-en-Y gastric bypass (RYGB) or sleeve gastrectomy (SG).

## Methods

The LABS-2 study enrolled adults with severe obesity undergoing bariatric surgery (2006-2009) at 10 US centers (NCT00465829). The institutional review boards at all 10 centers (University of Pittsburgh Medical Center [Pennsylvania]; Columbia University Medical Center, Weill-Cornell University Medical Center, and Valley Hospital [New York]; East Carolina University [North Carolina]; Neuropsychiatric Research Institute [North Dakota]; Oregon Health & Science University and Legacy Good Samaritan Hospital [Oregon]; and Virginia Mason Hospital and the University of Washington [Washington]) approved the study; participants provided written informed consent. Preoperative and annual postoperative assessments (except year 6) included a validated participant-reported UI questionnaire (UIQ) (eMethods in Supplement 1).^5^ Of 1829 LABS-2 study participants who underwent RYGB or SG, 1669 completed the UIQ preoperatively, of whom 1227 (73.5%) with long-term (≥5 years) follow-up are included in this cohort study, which followed the STROBE reporting guideline. The primary outcome was at least weekly UI prevalence. The frequency of UI episodes, the remission and incidence of UI, and the percentage weight loss were also evaluated. These outcomes and the statistical analyses, conducted in 2022, are detailed in the eMethods in Supplement 1. In brief, analyses were stratified by sex. Mixed models were used to estimate outcomes by year, to test time trends between 3 and 7 years (to evaluate stability during longer-term follow-up), and to compare outcomes at year 7 vs preoperative (to evaluate long-term change). Modeled percentages or mean values with 95% CIs are reported. All *P* values are 2-sided and reported to guide interpretation of results.^6^

## Results

Among women (n = 986), the preoperative median (IQR) age was 46 (38-54) years, and median (IQR) body mass index (BMI; calculated as weight in kilograms divided by height in meters squared) was 46 (42-52). The Figure, A, shows the observed UI episode frequency and at least weekly prevalence over time. Despite an increase in frequency and prevalence from 3 to 7 years after surgery, estimates were lower at 7 years vs preoperatively (eg, UI prevalence was 52% [95% CI, 48%-54%] preoperatively, 20% [95% CI, 18%-24%] at year 3, and 30% [95% CI, 26%-33%] at year 7). Time trends were similar for stress UI and urge UI, respectively (Table). From 3 to 7 years, UI remission decreased from 66% (95% CI, 61%-71%) to 56% (95% CI, 52%-63%); UI incidence increased from 6% (95% CI, 5%-10%) to 11% (95% CI, 9%-16%).

**Figure.**
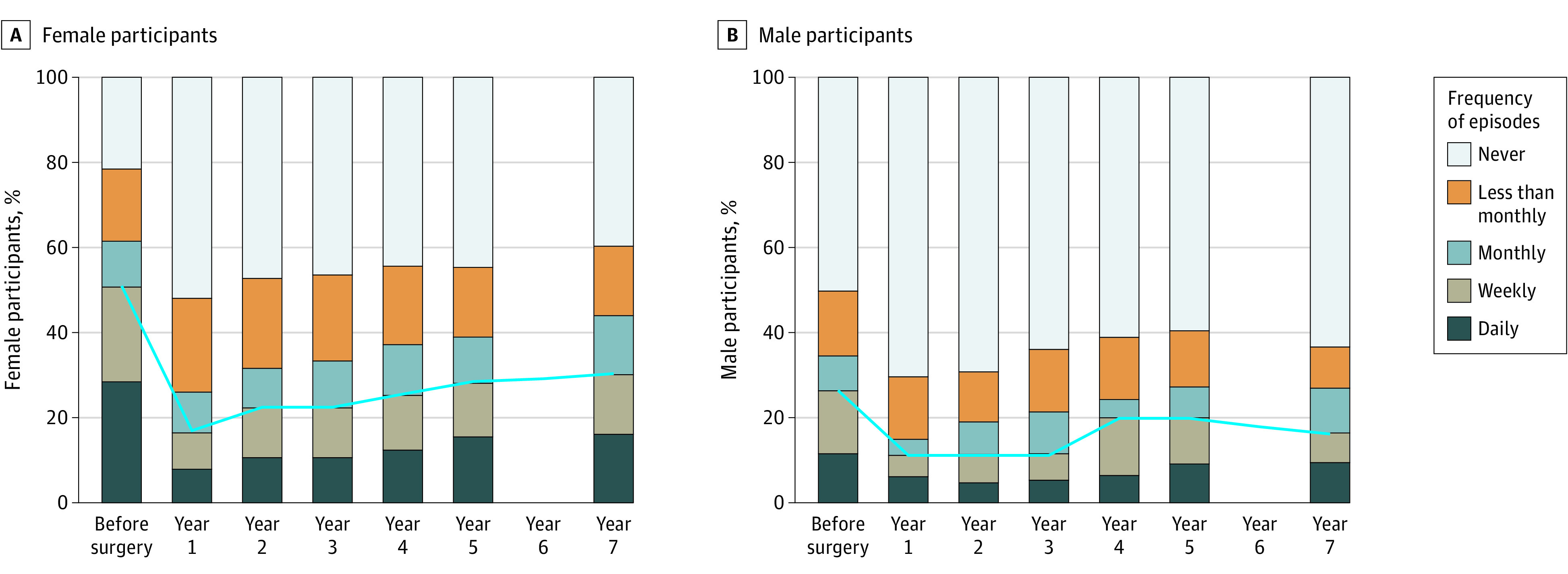
Observed Frequency of Urinary Incontinence Episodes by Time in Relation to Roux-en-Y Gastric Bypass or Sleeve Gastrectomy, by Sex The blue line in each panel indicates the prevalence of at least weekly urinary incontinence.

**Table.** Modeled Percentages of UI Frequency, Prevalence, Remission, and Incidence and Mean Percentage Weight Loss After RYGB or SG Stratified by Sex

Variable	No.	Years since surgery, model-based estimates, % or mean (95% CI)^a^							*P* value	
		Before surgery	Year 1	Year 2	Year 3	Year 4	Year 5	Year 7	Year 7 vs before surgery	Year 3 vs year 7^b^
**Female participants**										
Completed UIQ, No.		986	847	782	772	796	881	652	NA	NA
Frequency										
Never	986	24.4 (23.5-30.3)	50.4 (47.3-57.5)	46.5 (43.3-54.3)	45.2 (42.0-54.0)	43.3 (40.3-50.7)	43.8 (40.7-52.0)	39.4 (35.8-47.9)	<.001	<.001
<Monthly	986	16.0 (15.3-19.9)	20.7 (19.7-25.5)	19.7 (18.9-24.9)	18.9 (18.1-24.2)	17.8 (16.8-22.4)	15.6 (14.7-19.9)	15.6 (14.3-20.3)		
Monthly	986	10.1 (9.4-13.4)	9.1 (7.8-12.3)	8.6 (7.6-11.6)	10.1 (9.1-13.6)	10.9 (9.8-14.3)	10.0 (9.1-13.2)	12.5 (11.4-16.8)		
Weekly	986	20.2 (19.7-25.2)	10.0 (8.6-13.5)	12.3 (11.0-16.2)	12.9 (11.5-17.2)	13.7 (12.3-18.1)	13.0 (11.7-17.1)	14.5 (13.2-19.4)		
Daily	986	29.3 (26.7-35.6)	9.8 (7.9-13.5)	12.9 (11.3-17.4)	12.9 (11.4-17.4)	14.3 (12.7-18.9)	17.6 (16.3-22.6)	18.1 (16.7-24.0)		
Prevalence (≥weekly)										
Any type	986	51.5 (47.7-53.9)	14.7 (12.5-17.3)	20.2 (17.8-22.7)	20.4 (17.5-23.6)	23.9 (21.2-26.9)	26.5 (23.8-29.1)	29.5 (26.0-32.5)	<.001	<.001
Stress type	900	46.3 (41.8-48.6)	11.6 (9.7-14.4)	15.7 (13.3-18.4)	16.5 (14.1-19.3)	20.1 (17.5-22.8)	21.9 (19.2-24.7)	23.6 (20.6-26.5)	<.001	<.001
Urgency type	812	38.3 (33.7-40.6)	10.4 (8.6-12.9)	16.3 (14.1-19.7)	16.0 (13.6-19.0)	18.6 (16.1-21.6)	20.0 (17.7-23.1)	24.1 (20.7-27.2)	<.001	<.001
Remission^c^	498	NA	76.2 (71.3-80.4)	66.9 (61.6-71.9)	65.9 (60.5-70.8)	63.0 (58.5-68.0)	62.3 (57.4-67.4)	56.4 (51.7-63.1)	NA	.001
Complete remission^d^	498	NA	35.5 (30.3-40.0)	30.1 (24.9-35.1)	28.8 (24.1-33.5)	27.0 (22.6-31.9)	29.2 (24.3-34.1)	24.4 (19.6-29.5)	NA	.13
Incidence^e^	488	NA	3.0 (1.6-5.3)	6.0 (4.2-9.4)	6.3 (4.5-9.7)	8.4 (6.5-12.3)	10.4 (8.3-15.0)	11.4 (9.4-15.7)	NA	<.001
Percentage weight lost	986	NA	33.9 (33.5-34.4)	34.4 (33.8-35.1)	32.0 (31.4-32.7)	30.2 (29.6-30.9)	29.1 (28.5-29.8)	28.0 (27.2-28.7)	NA	<.001
**Male participants**										
Completed UIQ, No.		241	201	196	198	191	213	142	NA	NA
Frequency										
Never	241	47.4 (42.5-59.3)	67.4 (61.4-77.9)	64.9 (59.6-77.3)	62.0 (56.8-72.2)	56.7 (51.5-70.5)	56.8 (51.9-68.1)	59.9 (53.4-73.3)	.04	.24
<Monthly	241	14.7 (12.1-23.0)	14.9 (10.9-21.9)	12.0 (8.2-19.5)	15.6 (11.5-24.2)	14.2 (10.9-23.3)	13.1 (9.7-19.7)	9.0 (5.0-16.4)		
Monthly	241	7.5 (5.2-13.1)	3.9 (1.6-8.3)	7.4 (4.5-14.4)	10.3 (6.9-17.6)	4.5 (1.9-10.5)	7.7 (4.5-14.1)	10.3 (6.0-18.2)		
Weekly	241	15.6 (12.5-24.2)	6.5 (2.4-12.9)	9.6 (5.0-17.2)	6.3 (3.1-12.1)	16.7 (12.7-26.6)	11.5 (7.5-18.4)	8.7 (4.0-17.7)		
Daily	241	14.8 (11.3-25.0)	7.3 (3.7-14.1)	6.1 (2.5-12.4)	5.7 (2.1-12.5)	7.8 (4.0-15.1)	10.9 (6.8-18.7)	12.1 (6.3-23.0)		
Prevalence (≥weekly)										
Any type	241	24.8 (19.9-31.0)	9.1 (5.7-14.2)	9.4 (6.3-13.7)	10.2 (6.7-14.8)	17.4 (12.3-23.2)	18.4 (14.2-23.6)	14.8 (9.3-20.5)	.003	.006^f^
Stress type	188	6.4 (2.6-9.9)	3.9 (1.2-7.1)	2.3 (0.3-5.4)	2.1 (0.0-6.1)	5.9 (2.0-9.4)	5.2 (1.7-9.2)	7.7 (3.5-11.0)	.48	.059
Urgency type	228	19.8 (15.9-25.8)	5.8 (3.0-9.9)	6.8 (3.8-10.8)	7.2 (4.1-12.3)	13.4 (9.6-19.3)	12.7 (8.7-18.0)	11.2 (6.8-17.3)	.003	.02^f^
Remission^c^	63	NA	74.5 (62.7-86.5)	74.8 (63.0-85.9)	76.3 (63.2-88.5)	67.0 (51.8-81.1)	67.9 (53.6-80.5)	73.6 (57.6-86.9)	NA	.90
Complete remission^d^	63	NA	46.8 (33.0-59.9)	43.7 (29.5-59.6)	43.8 (29.2-56.9)	33.3 (19.0-48.2)	35.9 (21.9-51.0)	37.9 (23.9-52.8)	NA	.77
Incidence^e^	178	NA	3.0 (0.6-6.5)	3.3 (1.1-6.8)	5.3 (2.1-9.6)	11.1 (6.0-16.5)	13.1 (8.3-18.2)	11.9 (6.3-17.5)	NA	.03^f^
Percentage weight lost	241	NA	32.2 (31.2-33.2)	31.9 (30.7-33.2)	30.0 (28.6-31.1)	28.5 (27.1-29.9)	28.0 (26.7-29.4)	26.5 (25.0-27.9)	NA	<.001

Abbreviations: NA, not applicable; RYGB, Roux-en-Y gastric bypass; SG, sleeve gastrectomy; UIQ, urinary incontinence questionnaire.

^a^ All models were adjusted for preoperative age, smoking status, and site.

^b^ *P* values from linear trend test are reported if *P* ≥ .05 from quadratic trend.

^c^ Refers to change from prevalent UI (ie, weekly or more frequent UI episodes) preoperatively to less than weekly UI episodes at postoperative follow-up.

^d^ Refers to change from prevalent UI (ie, weekly or more frequent UI episodes) preoperatively to no UI episodes at postoperative follow-up.

^e^ Refers to change from less than weekly UI episodes preoperatively to prevalent UI (ie, weekly or more frequent UI episodes) at postoperative follow-up.

^f^ Indicates that quadratic trend test *P* value is reported.

Among men (n = 241), the preoperative median (IQR) age was 50 (41-57) years, and the median (IQR) BMI was 48 (43-53). The frequency of UI was similar from 3 to 7 years and lower at year 7 vs preoperatively (Figure, B; Table). The prevalence of UI increased from 10% (95% CI, 7%-15%) to 15% (95% CI, 9%-21%) from 3 to 7 years but was still lower vs preoperatively (25% [95% CI, 20%-31%]) (Table). From 3 to 7 years, UI remission was similar (from 76% [95% CI, 63%-89%] to 74% [95% CI, 58%-87%]); UI incidence increased from 5% (95% CI, 2%-10%) to 12% (95% CI, 6%-18%). Among both sexes, percentage weight loss decreased from 3 to 7 years (eg, from 32% [95% CI, 31%-33%] to 28% [95% CI, 27%-29%] in women) (Table).

## Discussion

In this large US cohort of women and men who underwent RYGB or SG, despite decreases across follow-up in initial postoperative improvements in UI and the increased risk of UI due to aging, UI frequency and at least weekly prevalence were substantially lower 7 years postoperatively vs preoperatively. Specifically, over half of women and men with preoperative UI experienced sustained UI remission, while postoperative incidence of UI was low. A limitation to this study was that it was observational without a control group; however, time trends of UI outcomes and percentage weight loss were similar. Durable improvement in UI is an important benefit of modern-day bariatric surgical procedures, which should be discussed with patients with severe obesity when making treatment decisions.

## Notes

Supplement 1.
eMethods.

eReferences.

Supplement 2.
Data Sharing Statement
