# Supplementary material for: Seven-Year Durability of Improvements in Urinary Incontinence After Roux-en-Y Gastric Bypass and Sleeve Gastrectomy
Source: JAMA Netw Open. 2022 Dec 9;5(12):e2246057. doi: 10.1001/jamanetworkopen.2022.46057 (PMC9856234; doi:10.1001/jamanetworkopen.2022.46057)
Supplement: Supplement 1. — eMethods. eReferences. [file jamanetwopen-e2246057-s001.pdf]

Supplemental Online Content

King WC, Hinerman AS, Subak LL. Seven-year durability of improvements in urinary incontinence after Roux-en-Y gastric bypass and sleeve gastrectomy. *JAMA Netw Open*. 2022;5(12):e2246057. doi:10.1001/jamanetworkopen.2022.46057

eMethods.

eReferences.

This supplemental material has been provided by the authors to give readers additional information about their work.

eMethods

LABS-2 participants completed a preoperative assessment and annual follow-up assessments for 7 years or until January 2015, whichever came first[1]. The year 6 assessment was brief and did not include the urinary incontinence questionnaire (UIQ).

On the UIQ participants were asked, "In the past 3 months, how often have you typically leaked urine, even a small amount?" Response options were "never, less than once per month, monthly (once or more each month), weekly (once or more each week), or daily (once or more each day)." Those answering daily or weekly reported the number of times per week that urine loss occurred, "with a physical activity like coughing, sneezing, lifting or exercise" (to measure stress-type UI), "with an urge or the feeling that you needed to empty your bladder but you could not get to the toilet fast enough" (to measure urgency-type UI), and for "other reasons" (to measure any other UI episodes). These UI questions were previously validated against voiding diaries,[1] the gold standard for assessment of UI frequency and type.

Because at least weekly UI is associated with reduced quality of life and increased treatment-seeking, participants with at least weekly UI of any type were considered to have prevalent UI. Participants were defined as having prevalent stress- or urgency-type UI if they reported stress-type or urgency-type UI, respectively, at least weekly.

Participants with prevalent UI preoperatively (N=498 women, 63 men) were evaluated for remission, defined as less than weekly UI at postoperative follow-up, and complete remission, defined as no UI episodes at postoperative follow-up. Participants without prevalent UI preoperative (N=488 women, 178 men) were evaluated for incident UI, defined as prevalent UI at postoperative follow-up. Percent weight loss was calculated as 100*[(preoperative weight – postoperative weight)/preoperative weight].

Analyses were conducted using SAS V.9.4 (SAS Institute Inc, Cary, NC). Analyses were stratified by sex. Data were assumed to be missing at random (i.e., the probability of missing depends only on the observed data) and all available observations were used. Outcomes were estimated by time point and differences in distributions of outcomes over time were tested using mixed models with a person-level random intercept and time (assessment) as a discrete conditional likelihood, controlling for preoperative factors related to missing follow-up data (site, age and

© 2022 King WC et al. *JAMA Network Open*.

smoking status) as fixed effects. Specifically, binary mixed models were used for prevalence, remission and incidence, ordinal mixed models for frequency, and generalized linear mixed models via maximum likelihood for percent weight loss. We made pairwise comparisons between the preoperative and last (year 7) assessments to assess long-term changes. Because we previously evaluated time trends through 3 years postoperative, we also evaluated stability during longer-term follow-up. This was done by limiting the dataset to years 3-7 and testing for linear and quadratic trends with time (days since surgery) as conditional likelihood. Modeled percentages or means with 95% confidence intervals and two-sided P-values are reported to guide interpretation of results[3].

eReferences

1. Belle SH, Berk PD, Courcoulas AP, et al. Safety and efficacy of bariatric surgery: Longitudinal Assessment of Bariatric Surgery. Surgery for obesity and related diseases : official journal of the American Society for Bariatric Surgery. 2007;3(2):116-126.
2. Bradley CS, Brown JS, Van Den Eeden SK, SchembriM, Ragins A, Thom DH. Urinary self-report questions: reproducibility and agreement with bladder diary. *Int Urogynecol J*. 2011;22(12):1565-1571.
3. Wasserstein RL, Lazar NA. The ASA Statement on P values: context, process, and purpose. *Am Stat.* 2016;70(2):129-133.

© 2022 King WC et al. *JAMA Network Open*.
