# Supplementary material for: Seven-Year Durability of Improvements in Urinary Incontinence After Roux-en-Y Gastric Bypass and Sleeve Gastrectomy
Source: JAMA Netw Open. 2022 Dec 9;5(12):e2246057. doi: 10.1001/jamanetworkopen.2022.46057 (PMC9856234; doi:10.1001/jamanetworkopen.2022.46057)
Supplement: Supplement 2. — Data Sharing Statement [file jamanetwopen-e2246057-s002.pdf]

Data Sharing Statement

King. Seven-Year Durability of Improvements in Urinary Incontinence After Roux-en-Y Gastric Bypass and Sleeve Gastrectomy. *JAMA Netw Open*. Published December 09, 2022. doi:10.1001/jamanetworkopen.2022.46057

Data
Data available: No

Additional Information
Explanation for why data not available: Data from the Longitudinal Assessment of Bariatric Surgery-2 study can be requested from the National Institute of Diabetes and Digestive and Kidney Diseases (NIDDK) Central Repository. Please see repository.niddk.nih.gov.
